# COVID-19 and psychiatric disorders: The impact of face masks in emotion recognition face masks and emotion recognition in psychiatry

**DOI:** 10.3389/fpsyt.2022.932791

**Published:** 2022-09-27

**Authors:** Andrea Escelsior, Maria Bianca Amadeo, Davide Esposito, Anna Rosina, Alice Trabucco, Alberto Inuggi, Beatriz Pereira da Silva, Gianluca Serafini, Monica Gori, Mario Amore

**Affiliations:** ^1^Applied Neurosciences for Technological Advances in Rehabilitation Systems (ANTARES) Joint Lab, Clinica Psichiatrica ed SPDC, Largo Rosanna Benzi, Genoa, Italy; ^2^Department of Neuroscience, Rehabilitation, Ophthalmology, Genetics, Maternal and Child Health (DINOGMI), Section of Psychiatry, University of Genoa, Genoa, Italy; ^3^IRCCS Ospedale Policlinico San Martino, Genoa, Italy; ^4^U-VIP Unit for Visually Impaired People, Fondazione Istituto Italiano di Tecnologia, Genoa, Italy

**Keywords:** COVID-19, emotion recognition, face masks, psychiatric disorders, happiness

## Abstract

Since the outbreak of the COVID-19 pandemic, reading facial expressions has become more complex due to face masks covering the lower part of people's faces. A history of psychiatric illness has been associated with higher rates of complications, hospitalization, and mortality due to COVID-19. Psychiatric patients have well-documented difficulties reading emotions from facial expressions; accordingly, this study assesses how using face masks, such as those worn for preventing COVID-19 transmission, impacts the emotion recognition skills of patients with psychiatric disorders. To this end, the current study asked patients with bipolar disorder, major depressive disorder, schizophrenia, and healthy individuals to identify facial emotions on face images with and without facial masks. Results demonstrate that the emotion recognition skills of all participants were negatively influenced by face masks. Moreover, the main insight of the study is that the impairment is crucially significant when patients with major depressive disorder and schizophrenia had to identify happiness at a low-intensity level. These findings have important implications for satisfactory social relationships and well-being. If emotions with positive valence are hardly understood by specific psychiatric patients, there is an even greater requirement for doctor-patient interactions in public primary care.

## Introduction

The impact and social consequences of the COVID-19 pandemic varied depending on factors such as social inequalities (1), age, gender (2), and the presence of medical (3) and psychiatric (4) conditions. Moreover, among individuals with mental health conditions, COVID-19 presents higher rates of complications, hospitalization, and mortality (5). The history of psychiatric illness confers a heightened vulnerability to disaster-related conditions (6).

Owing to the outbreak of the COVID-19 pandemic, the use of face masks has become somewhat widespread depending on different state laws and people's subjective attitudes (7). Although different beliefs about the effectiveness of this personal protective equipment, face mask has been widely adopted to reduce disease transmission (8, 9). Wearing face masks significantly impacts the human capacity to read facial expressions [for a comprehensive review, see (10)], making it more difficult to recognize people's emotions and their intensity (7, 11–14). The ancestral origin and crucial phylogenetic importance of the facial emotion recognition process in social interactions has been apparent since Darwin's first observations 150 years ago in his book “The Expression of the Emotions in Man and Animals” (15). His intuition has received further confirmations to date, due in particular to Paul Ekman's work (16). Reading facial expressions is an essential component of non-verbal communication in humans, together with head orientation (17), posture, body language (7) or characteristics of voice (18). While understanding affective expressions is a key social ability, its deficit is associated with severe difficulties in human interactions (19). Before the pandemic, studies reported that people had difficulty reading facial emotions when others were wearing some objects that cover parts of the face, for example: cardboard (20), a cap, or a scarf (21). More recently, studies on face masks reported that covering the lower part of the face altered the facial emotion reading (22), probably due to the constraint of focusing on the eye region compared to the mouth region (23, 24).

Mental illness conditions are often characterized by a different magnitude of impairments in social functioning and interpersonal interactions (25), linked to significant impairments in emotional expression reading. Participants with psychiatric disorders showed different degrees of impairment in facial emotion recognition (26). Such impacts on the emotional reading of faces mainly depend on shared alterations of dimensions such as mood (27), social cognition (28), or metacognition (29) among mental illnesses. Given the well-known disadvantages in social interactions of participants who present with a mental illness (Wild and Kornfeld 2021), the current study aims to assess how the widespread use of face masks impacts the emotion recognition skills in patients with psychiatric disorders.

For this purpose, we asked a group of participants with bipolar disorder (BD), major depressive disorder (MDD), schizophrenia (SZ), and a healthy control (HC) group to identify facial emotions on images with and without face masks. Our study tested varying intensities of facial expressions to investigate mild levels of impairment and recall more realistic facial configurations.

## Methods

### Sample

The current study recruited twenty-eight HC, 15 participants with BD, 20 participants with MDD, and 13 participants with SZ (see Table 1). The study excluded one participant with MDD and two participants with BD from the analyses because they were identified as outliers (i.e., a score in at least one task differing more than two standard deviations from the group's mean score). Thus, the remaining group was comprised of 28 HC (mean age ± standard deviation = 41.7 years old ± 11.8; females = 23), 13 participants with BD (39.6 years old ± 11.8; females = 5) 19 participants with MDD (48.4 years old ± 21.8; females = 15), and 13 participants with SZ (48.1 years old ± 8.5; females = 6). Groups were age-matched (*F*_(3, 69)_ = 1.5, *p* > 0.05). The study included a power analysis based on previously published studies testing participants' ability to recognize emotion with and without masks among healthy adults (22), indicating a minimum of 13 participants was necessary to reach a power of 0.85 (two-tailed *t*-test, Cohen's d = 1.2, α = 0.05).

**Table 1:** Details of participants for the four groups involved in the study.

**Group**	**Sample size**	**Age**	**Gender**
	***number***	***mean ±standard deviation***	***number***
Healthy control (HC)	28	41.7 ± 11.8 years old	23 F, 5 M
Bipolar disorder (BD)	13	39.6 ± 11.8 years old	5 F, 8 M
Major depressive disorder (MDD)	19	48.4 ± 21.8 years old	15 F, 4 F
Schizophrenia (SZ)	13	48.1 ± 8.5 years old	6 F, 7 M

In gender, F, female and M, male.

All psychiatric patients were recruited from the Psychiatric Unit of San Martino Hospital in Genoa, and they were hospitalized while testing was occurring. The study recruited typical participants from the general population using advertising on social media and personal newsletters. Moreover, they underwent a clinical interview to exclude the presence of lifetime or current psychiatric disorders. Participants did not receive incentives of any kind for participating in the study. The Ethical Committee of IRCCS Ospedale Policlinico San Martino approved the study, and all participants gave their written informed consent.

### Experimental paradigm

To investigate how face masks affect emotion recognition in psychiatric patients during hospitalization, we administered an internet-based questionnaire *via* smartphone. The questionnaire required participants to identify facial emotions on images with and without facial masks. Specifically, we replicated the paradigm that researchers previously used to test the effects of face masks on emotion recognition during childhood (22). This consisted of a standardized verbal-response test based on selecting an emotion's label (forced-choice) as a means to describe static pictures of human facial configurations. Such a choice favored the repeatability of the task and simplified the test administration to overcome the difficulties related to hospitalization and social distancing rules.

**Figure 1:**
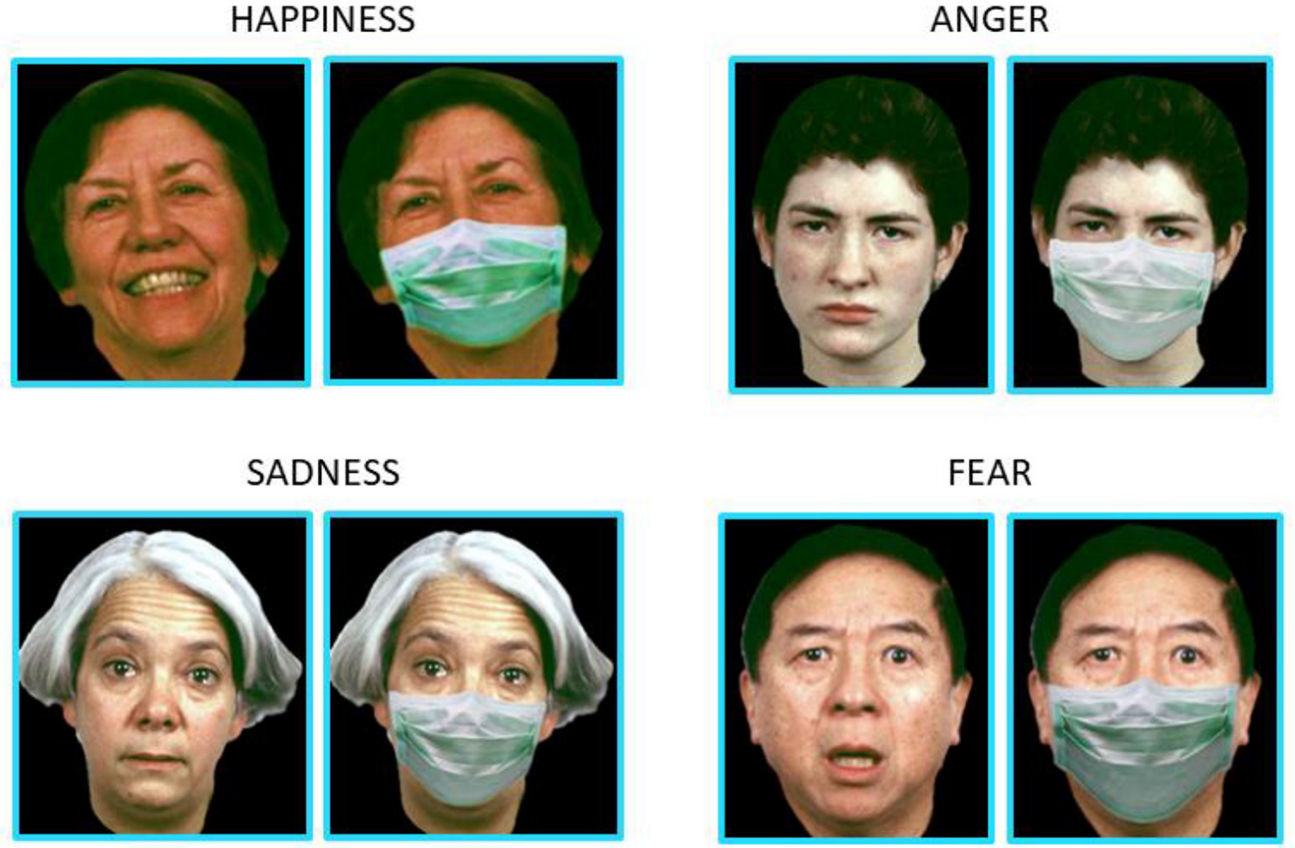
Examples of low-intensity facial configuration with and without face masks for happiness, anger, sadness, and fear. Face images were obtained with permission from the ER-40 color emotional stimuli public database (30, 31).

**Figure 2:**
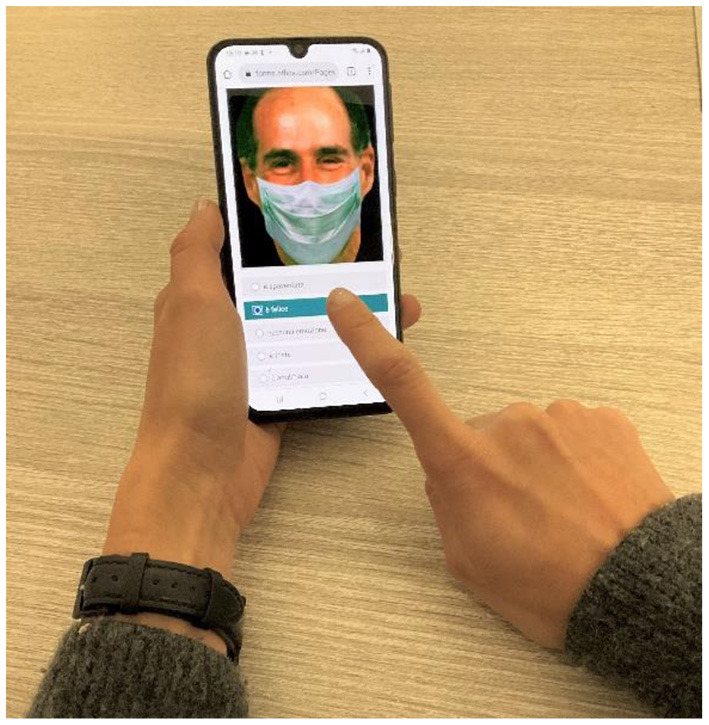
Experimental procedure. We asked participants to identify the correct facial emotion by choosing between five possible randomized options: happy, sad, fearful, angry, and neutral. Each face was displayed on the screen of personal smartphones for as long as it took to respond by holding an index finger against the touch screen. The study obtained face images with permission from the ER-40 color emotional stimuli public database (30, 31).

The task was structured in sequential blocks, showing first a block of pictures with facial masks, followed by a block of mask-free images. A total of 40 adult face pictures were presented in randomized order, including four repetitions of four facial emotions (happiness, sadness, fear, anger) with two levels of intensity (Low, High), in addition to a neutral facial expression that was presented 8 times to each participant. Figure 1 offers example images of happiness, sadness, fear and anger, with a low level of intensity. The original and modified pictures were obtained from the ER-40 color emotional stimuli database (30, 31), developed for the validated ER-40 test for facial emotion recognition (32, 33). A web designer modified pictures from the original database *ad hoc*, creating and adding realistic face masks for the set of images containing masks. We asked participants to identify their facial emotions by choosing five possible randomized options: happy, sad, fearful, angry, and neutral (see Figure 2).

To control for face mask exposure, the test occurred one year following the first lockdown's end in Italy (May 2021). Patients performed the test autonomously under the supervision of a clinical doctor, while typical participants performed it without supervision (the participants received specific written instructions, including the instruction to perform the task without any help). We did not impose time limits to provide answers.

### Data analyses

For data analysis, we calculated performance as a percentage of correct responses with and without the masks. Performance was not normally distributed for one group (Shapiro-Wilk normality tests: HC: W = 0.91, *p* < 0.01; BD: W = 0.95, *p* > 0.05; MDD: W = 0.96, *p* > 0.05; SZ: W = 0.97, *p* > 0.05); we then ran ANOVAs based on permutation tests and permutation *t*-tests. We used the *aovp* function (*lmPerm* package) and the *perm.t.test* function (*MKinfer* package) in R to compute the analysis. First, for each emotion separately (i.e., Happiness, Sadness, Fear, Anger), we ran an ANOVA based on permutation tests with mask presence (i.e., Mask, NoMask) and intensity level of emotions (i.e., Low, High) as within-subject factors and group (i.e., HC, BD, MDD, SZ) as between-subject factors. Considering there were no significant interactions between mask presence x intensity x group for anger, fear, and sadness but only for happiness, we focused subsequent analyses on emotional valence. We marked happiness as positive emotional valence, while grouping sadness, fear and anger into negative emotional valence. We thereby ran an ANOVA based on permutation tests with group (i.e., HC, BD, MDD, SZ) as between-subject factor, condition (i.e., Mask, NoMask), the intensity level of emotions (i.e., Low, High) and valence (i.e., Positive, Negative) as within-subject factors. We carried out follow-up ANOVAs using permutation tests and *post hoc* comparisons, applying Bonferroni correction to the results.

The intensity was absent as a variable for neutral faces. For the neutral expression, we performed a separate ANOVA based on permutation tests that considered only mask presence (i.e., Mask, NoMask) and group (i.e., HC, BD, MDD, SZ). Moreover, we computed confusion matrices to investigate the response distribution among different emotions with masks for each group.

## Results

Results showed that face masks always negatively impact the human ability to recognize emotions from facial configurations, but in the current study, this was particularly true for patients with MDD and SZ who were asked to recognize low-intensity images with positive valence. Indeed, the ability of patients with MDD and SZ to infer happiness when happy facial configurations were relatively subtle is drastically influenced by face masks.

When considering each emotion separately, the interaction between mask presence x intensity x group appeared significant only for happiness, which offered the opportunity to group fear, anger and sadness and analyse them together based on their negative valence. Specifically, the interaction was insignificant for anger [*F*_(3, 207)_ = 0.1, *p* > 0.05, Iter = 51], sadness [*F*_(3, 207)_ = 0.7, *p* > 0.05, Iter = 556], and fear [*F*_(3, 207)_ = 0.2, *p* > 0.05, Iter = 424]. Subsequently, the ANOVA considering mask presence, group, valence and level of intensity demonstrated a significant main effect of mask presence [*F*_(1, 1, 067)_ = 54.7, *p* < 0.01, Iter = 5,000], group [*F*_(3, 69)_ = 6.5, *p* < 0.01, Iter = 5,000], valence [*F*_(1, 1, 067)_ = 184.7, *p* < 0.01, Iter = 5,000] and level intensity [*F*_(1, 1, 067)_ = 54.7, *p* < 0.01, Iter = 5,000]. Moreover, this analysis revealed a significant interaction between the involved factors [mask presence x group x valence x level of intensity: *F*_(3, 1067)_ = 2, *p* < 0.01, Iter = 5,000].

**Figure 3:**
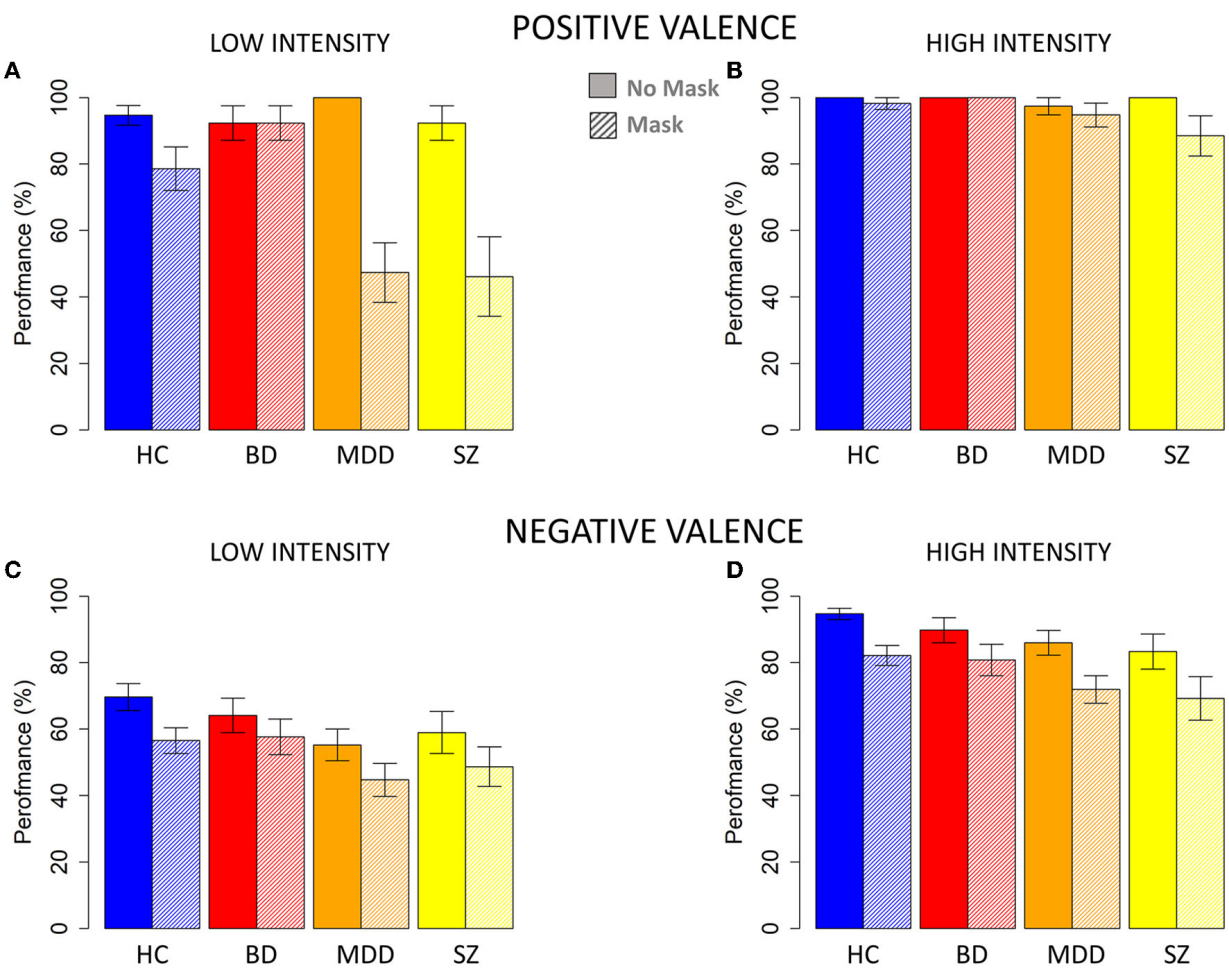
Percentage of correct responses without and with face masks for each group. **(A)** Performance for images with low-level positive valence. **(B)** Performance for images with high-level positive valence. **(C)** Performance for images with low-level negative valence. **(D)** Performance for images with high-level negative valence. HC, healthy control; BD, patients with bipolar disorder; MDD, patients with major depressive disorder; SZ, patients with schizophrenia. Filled and shaded color bars represent images without and with face masks, respectively. The standard error of the mean (SEM) is reported.

Concerning the emotion with positive valence (i.e., happiness, Figure 3 top), the follow-up analyses demonstrated a significant interaction between mask presence x group x level of intensity [*F*_(3, 276)_ = 4.8, *p* < 0.05, Iter = 5,000], allowing us to separately analyse the two levels of intensity. For high-intensity emotions with positive valence (Figure 3A) only a significant main effect of mask presence emerged [*F*_(1, 69)_ = 3.8, *p* < 0.01, Iter = 2,865], while there was no significant effects for group [*F*_(3, 69)_ = 2.1, *p* > 0.05, Iter = 724] and the interaction mask presence x group [*F*_(3, 69)_ = 1.6, *p* > 0.05, Iter = 1,377]. Instead, for low-intensity emotions with positive valence (Figure 3B), the interaction between mask presence and group was statistically significant [*F*_(3, 69)_ = 6.6, *p* < 0.01, Iter = 5,000]. *Post hoc* permutation *t*-tests showed masks' presence reduced a participant's ability to recognize emotions with positive valence for HC [*t*_(37.8)_ = −2.2, *p* < 0.01, Iter = 5,000], MDD patients [t_(18)_ = −5.9, *p* < 0.01, Iter = 5,000], and SZ patients [t_(16.4)_ = −3.5, *p* < 0.01, Iter = 5,000], but not BD patients [t_(24)_ = 0.0001, *p* > 0.05, Iter = 5,000]. Moreover, while patients and control participants performed similarly without masks [for HC vs. BD: t_(20.2)_ = 0.3, *p* > 0.05, Iter = 5,000; for HC vs. MDD: t_(27)_ = −1.8, *p* > 0.05, Iter = 5,000; for HC vs. SZ: t_(20.2)_ = 0.4, *p* > 0.05, Iter = 5,000; for BD vs. MDD: t_(12)_= 1.5, *p* > 0.05, Iter = 400; for BD vs. SZ: t_(24)_ = 0.0001, *p* > 0.05, Iter = 5,000; for MDD vs. SZ: t_(12)_ = 1.5, *p* > 0.05, Iter = 400], analyses showed some differences between groups when masks covered half of one's face. Specifically, MDD patients performed worse than HC participants [t_(35.5)_ = 2.8, *p* < 0.01, Iter = 5,000], and BD patients [t_(27.5)_ = −4.3, *p* < 0.01, Iter = 5,000]. Similarly, SZ patients performed worse than HC [t_(19.4)_ = 2.4, *p* < 0.01, Iter = 5,000] and BD patients [t_(16.4)_ = 3.5, *p* < 0.01, Iter = 5,000]. HC participants and BD patients had similar performance with masks [t_(37.8)_ = −1.6, *p* > 0.05, Iter = 5,000], as did MDD and SZ patients [t_(24.1)_ = 0.08, *p* > 0.05, Iter = 5,000].

For emotions with negative valence (Figure 3 bottom), the interaction mask presence x group x level of intensity was insignificant [F_(3, 791)_ = 0.07, *p* > 0.05, Iter = 51]. The analysis showed an overall decrease of performance correlated with mask presence [F_(1, 791)_ = 54.7, *p* < 0.01, Iter = 5,000], low level of intensity [F_(1, 791)_ = 184.7, *p* < 0.01, Iter = 5,000] and group [F_(3, 69)_ = 6.5, *p* < 0.01, Iter = 5,000]. As Figures 3C,D indicate, the percentage of corrected responses gradually decreased independent of intensity level among HC participants, patients with BD, patients with MDD, and patients with SZ.

When analyzing neutral expressions, we observed that masks similarly affected the performance of all participants, with no differences between groups. Indeed, a main effect of mask presence emerged from the ANOVA on performance [F_(1, 69)_ = 8.5, *p* < 0.01, Iter = 4,913] but not a main effect of group [F_(3, 69)_ = 1.9, *p* > 0.05, Iter = 432] or an interaction between mask presence and group [F_(1, 69)_ = 1.9, *p* > 0.05, Iter = 962].

**Figure 4:**
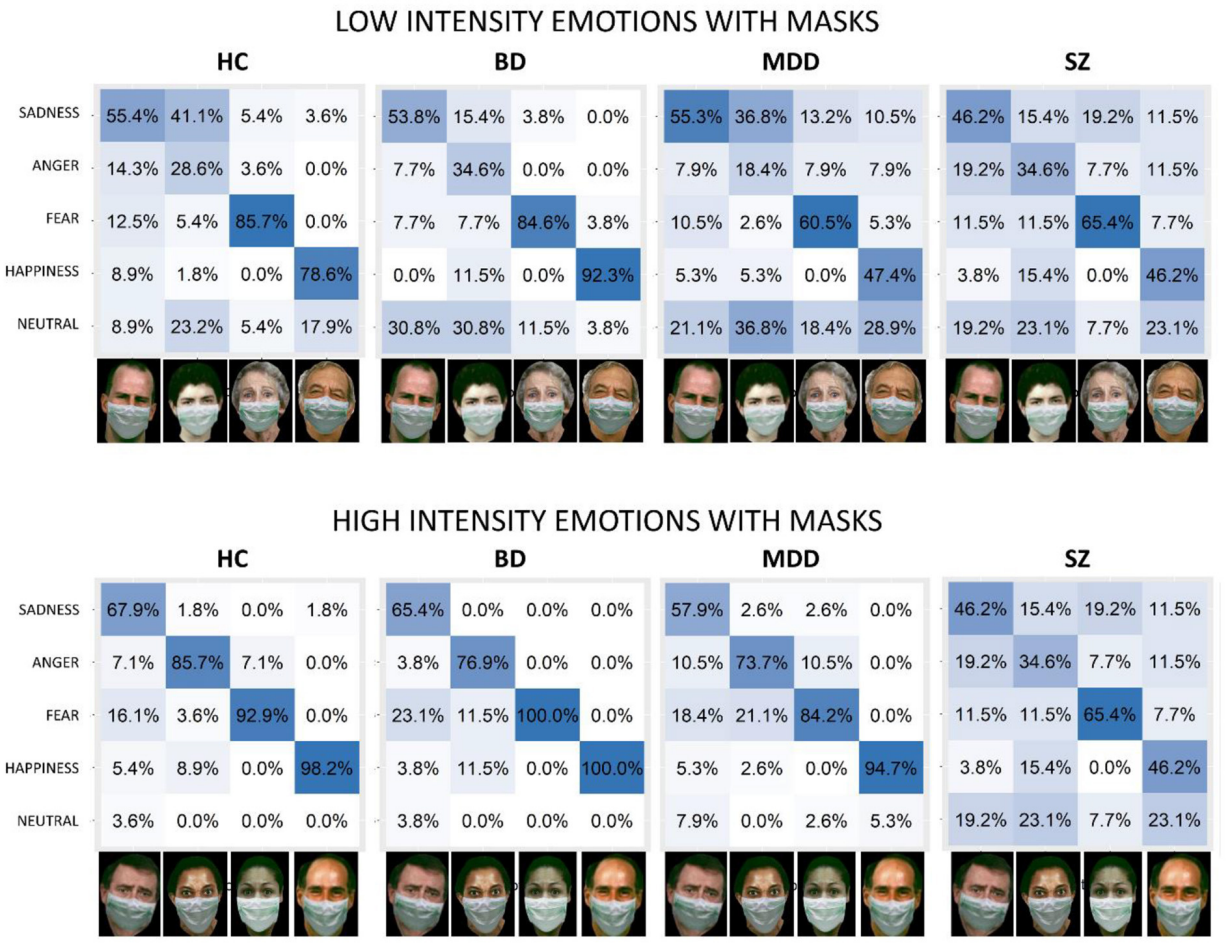
Confusion matrices for emotion inference from low-intensity **(bottom)** and high-intensity **(top)** facial configurations with face masks for all groups. The x-axis shows the presented stimuli. The y-axis shows the emotions perceived by participants. Columns report the percentage of responses for each emotion. HC, healthy control; BD, patients with bipolar disorder; MDD, patients with major depressive disorder; SZ, patients with schizophrenia. Face images were obtained with permission from the ER-40 color emotional stimuli public database (30, 31).

Figure 4 presents response distribution among different emotions with masks, indicating the matrices of confusion for low and high levels of intensity emotions for HC, BD, MDD, and SZ individuals, respectively. We excluded the responses to neutral expressions as they do not involve two levels of intensity. All participants confused the correct emotion with other emotions more often when the mask was present. For all groups, confusion increased in the low-intensity condition, and this was especially true for MDD and SZ patients. The most challenging emotion to recognize was anger in line with (30, 33), which participants typically recognized as a neutral expression or sadness. Participants regularly misrecognized happy faces covered with masks as neutral expressions, while also confusing sad faces covered with masks with neutral expressions and all the other emotions.

## Discussion

In this study, we investigated whether psychiatric patients, and particularly those affected by BD, SZ, and MDD, have more difficulties than healthy people recognizing facial emotions with a part of the face covered by a face mask. We demonstrated that using face masks overall reduces recognition performance across all individuals. Moreover, hiding the lower part of the face with face masks specifically impairs the recognition of subtle happy faces for SZ and MDD.

We replicated literature findings of a negative effect in recognizing facial expressions due to face masks (14, 22, 34). As expected, in specific cases, the difficulty is much higher for psychiatric patients. Indeed, impairment is particularly intense for positive faces with low-intensity emotional valence: face masks critically altered the chances of MDD and SZ participants recognizing happiness when it is slight. These results further confirm the importance of the mouth region in recognizing this emotion (23, 35). The reason for the drop in performance in MDD and SZ individuals when they must recognize low-intensity happy faces with masks may result from the negative symptoms these groups of patients share. An inverse association has been shown between the accuracy in recognizing happy expressions and depression severity (36). As well, depression drives people to bias facial expressions toward negative emotions like sadness, thus under-recognizing happy facial expressions in comparison with healthy participants (36). Given the crucial importance of the mouth to infer the facial expression of happiness (37), it is reasonable to hypothesize that when a mask covers this region there results in a real struggle to recognize happiness in the presence of negative symptoms. A ceiling effect for low-intensity happiness without masks might also underly the lack of differences between the two groups of patients and, if this is the case, this limits the generalizability of our results to real-life situations. Indeed, for high-intensity positive emotions with and without masks, and low-intensity positive without masks, we did not observe significant differences between the groups (SZ, MDD, BD, and HC). The lack of differences between patients and controls in these conditions is likely due to the dataset of images that involves very clear stimuli concerning positive emotions, evoking ceiling effects (33). In contrast to the commonly used emotion recognition tasks, we chose to use an easier task to emphasize the difference between the masked vs. non-masked conditions. Another non-mutually exclusive hypothesis is that the presence of masks during the year of COVID-19 before our experiment helped patients in their overall ability to recognize emotions. We can speculate that focusing only on the eyes during the pandemic improved skills to recognize facial expressions; when only half the face is available, patients generally had to learn to be more responsive to eye cues. If this is the case, patients still face difficulties when masks cover part of the face but became more similar to HC when the whole face was visible. Further research is necessary to validate this latter hypothesis. The fact that the performance drastically decreases when low-intensity happy expressions are covered with masks stresses the importance of the mouth region in recognizing happiness when negative symptoms are present.

As for emotion with negative valence (i.e., fear, anger, and sadness), we observed that the presence of masks similarly impacted the performance of all participants. We hypothesize that the deficit associated with the mask is present in all groups but not particularly impairing. This is because recognizing anger and fearful expressions largely requires information from the eyes (38–40). In line with previous results, performance significantly decreases with reduced intensity (33) and, independent of intensity level, the percentage of corrected responses was higher for HC and decreased for BD, followed by MDD and SZ. This agrees with the overall difficulty of psychiatric patients in reading facial emotions. For instance, a recent review by Krause et al. (36) stresses the existence of a broad facial emotion recognition deficit in individuals suffering from MDD. Among participants affected with BD, available evidence accounted for a global or selective facial expression recognition deficit in euthymic participants, or during the active phase of illness in nearly 2/3 of the available studies (41). Patients affected with BD are significantly less accurate when it comes to recognizing facial emotions but particularly fear (42, 43). Since the first episode, psychotic patients displayed a global impairment in recognizing facial affective expressions, and in particular negative emotions like fear and anger (28). Similarly, participants with SZ are generally insensitive or misrecognized negative emotions such as sadness, fear, and anger (44) while also being more likely to misinterpret happy faces (45). In SZ patients, the abnormal face processing seems to depend on a faulty structural encoding of faces (45, 46) and on the tendency to visually scan features of the face that are not important in the expression of a specific emotion (47).

Regarding neutral expressions, our study agrees with previous findings that reported a certain difficulty in recognizing the neutral expression, a difficulty accentuated when the face mask is worn (39, 48).

To conclude, the outcome of our work is that wearing a face mask makes each facial expression much more complex to recognize, regardless of the underlying psychological disorder. However, when the face mask is on, difficulties in recognizing happy facial emotions become even more severe for SZ and MDD patients. Nevertheless, this study has some limitations for which to account when interpreting the results: samples were relatively small and unequal in terms of the number of participants; the visual input includes different positions of the head, head tilt, etc. beyond information about facial emotions; all patients were hospitalized at the moment of testing, questioning the generalizability of results when it comes to applying them to non-hospitalized people suffering from mental health conditions; the experimental setup challenges the ecological validity of a computerized test vs. real-life situations. Furthermore, future studies should address possible effects resulting from a lack of gender-matched samples. Indeed, emotion recognition is gender specific, with females known to better perform (49–51), and females predominated our sample of MDD in line with the skewed gender ratio for this psychiatric condition (51). Females also predominated the HC group because we purposefully matched it with the gender bias of the MDD group. Moreover, we cannot completely rule out other potential confounds, such as visual acuity, previous experience with this kind of paradigm, or personality traits. Although further research is necessary, our findings retain important clinical implications. They may explain why the use of portrait photos with smiling faces positively affects patients' perceptions of healthcare staff (52). Additionally, the impairment of positive implicit communication might contribute to misinterpretation of other intentions and emotions during social relationships (53), with negative consequences on clinical interactions with patients of mental health workers such as psychiatrists, psychologists, psychiatric rehabilitation technicians, or nurses. Moreover, recognizing emotions with positive valence is crucial for the patient's social interactions and well-being in general.

## Data availability statement

The raw data supporting the conclusions of this article will be made available by the authors, without undue reservation.

## Ethics statement

The studies involving human participants were reviewed and approved by Ethical Committee of IRCCS Ospedale Policlinico San Martino. The patients/participants provided their written informed consent to participate in this study.

## Author contributions

AE, MBA, DE, and BP collected the data and organized the database. MBA performed the statistical analysis. AE, MBA, and AR wrote the first draft of the manuscript. AT helped to revised the manuscript. All authors contributed to the conception and design of the study, revising, reading, and approving the submitted version.

## Funding

This research was supported by the joint lab ANTARES between the Unit for Visually Impaired People (IIT) and the Section of Psychiatry of the Department of Neuroscience, Rehabilitation, Ophthalmology, Genetics, Maternal and Child Health (DINOGMI) of the University of Genoa. This work was developed within the DINOGMI Department of Excellence of MIUR 2018-2022 (Law 232; 2016).

## Conflict of interest

The authors declare that the research was conducted in the absence of any commercial or financial relationships that could be construed as a potential conflict of interest.

## Publisher's note

All claims expressed in this article are solely those of the authors and do not necessarily represent those of their affiliated organizations, or those of the publisher, the editors and the reviewers. Any product that may be evaluated in this article, or claim that may be made by its manufacturer, is not guaranteed or endorsed by the publisher.
